# Direct and indirect climate controls predict heterogeneous early-mid 21^st^ century wildfire burned area across western and boreal North America

**DOI:** 10.1371/journal.pone.0188486

**Published:** 2017-12-15

**Authors:** Thomas Kitzberger, Donald A. Falk, Anthony L. Westerling, Thomas W. Swetnam

**Affiliations:** 1 Laboratorio Ecotono, CONICET–INIBIOMA, Universidad Nacional del Comahue, Quintral, Bariloche, Argentina; 2 University of Arizona, Laboratory of Tree-Ring Research, Tucson, AZ, United States of America; 3 University of Arizona, School of Natural Resources and the Environment, Environment and Natural Resources Building, Tucson, AZ, United States of America; 4 Sierra Nevada Research Institute, University of California, Merced, California, United States of America; Universite du Quebec a Chicoutimi, CANADA

## Abstract

Predicting wildfire under future conditions is complicated by complex interrelated drivers operating across large spatial scales. Annual area burned (AAB) is a useful index of global wildfire activity. Current and antecedent seasonal climatic conditions, and the timing of snowpack melt, have been suggested as important drivers of AAB. As climate warms, seasonal climate and snowpack co-vary in intricate ways, influencing fire at continental and sub-continental scales. We used independent records of seasonal climate and snow cover duration (last date of permanent snowpack, LDPS) and cell-based Structural Equation Models (SEM) to separate direct (climatic) and indirect (snow cover) effects on relative changes in AAB under future climatic scenarios across western and boreal North America. To isolate seasonal climate variables with the greatest effect on AAB, we ran multiple regression models of log-transformed AAB on seasonal climate variables and LDPS. We used the results of multiple regressions to project future AAB using GCM ensemble climate variables and LDPS, and validated model predictions with recent AAB trends. Direct influences of spring and winter temperatures on AAB are larger and more widespread than the indirect effect mediated by changes in LDPS in most areas. Despite significant warming trends and reductions in snow cover duration, projected responses of AAB to early-mid 21^st^ century are heterogeneous across the continent. Changes in AAB range from strongly increasing (one order of magnitude increases in AAB) to moderately decreasing (more than halving of baseline AAB). Annual wildfire area burned in coming decades is likely to be highly geographically heterogeneous, reflecting interacting regional and seasonal climate drivers of fire occurrence and spread.

## Introduction

Wildfire is emerging globally as a key process mediating vegetation dynamics and anthropogenically altered biosphere-atmosphere carbon exchanges [1–5]. Although climatic variability is a dominant factor affecting the occurrence of large wildfires, overly simplistic views that warming will inexorably increase fire occurrence globally are being challenged as our understanding increases of the context-specific interplay of climate, biota, human activities and fire [6–8].

Future spatial and temporal combinations of increased temperatures, extended droughts, reorganized patterns of water availability, ignitions and vegetation feedbacks are likely to lead to a rearrangement of *pyrogeography*, the global distribution of fire in relation to its driving forces [7–10]. For example, higher temperatures and dry conditions favor fire spread, but these same conditions may also decrease plant growth and thus reduce production of flammable fuels [11]. Geographically-explicit predictions of fire potential are still emerging due to our limited understanding of the variability of biophysical controls on fire and vegetation growth among biomes, as well as within biomes over mesoscale topographic and climatic gradients [3], [12–16].

Weather conditions at local and regional scales during the fire season are dominant factors influencing fuel flammability and annual area burned [17–20]. Warmer temperatures in concert with drought decrease fuel moisture, increase lightning ignitions, and lengthen the period over which fires occur [21], [22]. At regional scales, the occurrence of severe fire weather is regulated strongly by seasonal climatology as reflected in synoptic-scale atmospheric circulation [23–26]. As a consequence, the potential impacts of seasonal weather on fire behavior and extent are spatially heterogeneous, for several reasons. Growing season temperature influences water balance deficits (PET-AET)–an important variable controlling annual area burned by regulating biomass and fuel moisture–variably across climates and ecosystems [22], [26–28]. Moreover, fire regimes in various vegetation types are limited by multiple factors, such as fuel quantity, structure or flammability, depending on the dominant factor limiting ignition and propagation, as well as spatially heterogeneous effects of previous fires [29–32].

In addition to fire-season weather, antecedent temperature and precipitation over prior months and years regulate fire frequency and extent to varying degrees across ecosystem types. These effects are especially strong where winter and spring moisture are limiting to growth and conditioning of fine fuels prior to fire season [26], [33], [34]. Ecosystems will thus generate differential responses in vegetation growth in response to warming and changes in precipitation, resulting in significant differences in fuel types and loads under altered climate [7], [35]. For example, although warming may increase area burned under extreme warming/drying conditions in more productive, less fire-prone ecosystems due to increased fuel desiccation, it may produce only modest changes or even decreased area burned in less productive, fuel limited ecosystems by restricting fuel production [3], [6], [15].

The timing of snowpack melt has been proposed as a primary mechanism that may influence fuel moisture and wildfire occurrence, including annual area burned at high elevations of temperate regions or high latitudes [27], [36]. Late winter/spring warming may increase fire activity if earlier snowmelt and runoff cannot compensate for peak evaporative water demand of ecosystems, thus inducing fuel desiccation at large scales [37], [38]. Although the timing of snowpack melt has been linked to increased fire activity, the relative contributions of snowpack duration (e.g., via deep wetting of soils, dead fuels, and thorough hydration of living fuels during the spring) and warmer spring temperatures (e.g., via increased evaporative demand) to fire activity are incompletely understood. Decreased water supply and increased evaporative demand may both contribute to increased fire activity, especially in warmer climates where snowpack declines before spring evaporative demands begins [39]. Thus, the relative importance and effects of spring warming on water supply and demand are spatially variable across ecosystems, generating geographically complex net outcomes of winter/spring warming on fire activity that need to be elucidated mechanistically. Statistical relationships among snowmelt timing, temperature and fire activity commonly show high degrees of collinearity between predictors, hindering the decoupling of these influences. Independent, spatially explicit climate and snowpack duration records are thus needed to assess the complex interrelationships and probable covariance between climate, snowpack, and fire occurrence over regional to continental scales.

Annual area burned (AAB) is a key measure of fire regimes, integrating fire size, frequency, and interannual climatic controls [13], [38], [40], [41]. Burned area is also a key multiplier of landscape-climatic interactions including pyrogenic carbon emissions [42]. Projections of future annual area burned based on a range of emission and climate scenarios have been prepared for some areas, indicating likely increases in AAB for the interior northwest of US, the northern US Rockies, and the southwestern US [6], [14], [28], [43], [44]. For other regions (e.g. boreal forests) projections of AAB with climate change range from increases, no change, to decreases in AAB, suggesting greater uncertainty in areas where warming may create counteracting effects (e.g. heterogeneous effects of precipitation, lagged effects on productivity, alleviation of spring drought; [7], [16]. In North American boreal forests, some authors argue that warmer fire seasons along with anthropogenic influences will generate higher fire activity [18], [19], whereas others assert that the recent upward trend in fire activity is heterogeneous over the entire biome and is relatively modest compared to a downward trend in fire activity starting in the 19^th^ century [20], [45–47].

To understand potentially heterogeneous responses of fire regimes to changes in snowpack and seasonal climate over broad geographic scales, we applied cell-based statistical modeling of seasonal climate controls on annual area burned over western and boreal North America. Our objectives were to: (1) identify key how seasonal and interannual climate controls on spatial variation in AAB at continental scales; (2) distinguish direct influences of warming on AAB through fuel desiccation (demand side) from indirect effects mediated by precipitation or the change in state of water during the cold season (e.g. snowpack duration) that regulates water availability to plants (supply side); (3) produce near-term (next 1–3 decades) spatially explicit projections of change in AAB under a conservative scenario of climate change; and (4) validate statistical AAB predictions with AAB trend extrapolations using a split-sample model procedure [48].

## Methods

### Data sources

*Fire*. For model development we compiled point fire location and area burned data from lands in the protection responsibility of Federal land management agencies in the western US (WA, OR, CA, MT, ID, ND, WY, UT, CO, NV, AZ and NM) forest fires >200 ha (1972–2004) (http://fam.nwcg.gov/fam-web/weatherfirecd/, see [27] and [44] online supplements), the Canadian National Fire Database (NFDB, http://cwfis.cfs.nrcan.gc.ca/ha/nfdb) and the 1939–2006 Alaska Large Fire Database, Alaska Interagency Coordination Center AICC [49], http://afsmaps.blm.gov/imf_firehistory/imf.jsp?site=firehistory).

For a common period 1972–2004 each fire was assigned to a 1x1° grid cell (domain 52–170°W, 25–72°N); a total of 1562 cells were evaluated. Annual area burned (AAB) was calculated for each cell over the period of record. Because some fires near cell boundaries could have crossed to neighboring pixels, a 5% Gaussian filter was applied to each AAB raster layer, so that the four immediate cells each received 1% of the focal cell’s AAB and the four diagonal cells received each the remaining 0.25%. AAB was log-transformed to ensure normality of residuals. For model verification we used state/province-integrated AAB data for the period 1972–2004 and updated it for the period 2005–2015 from the National Interagency Fire Center historical year-end fire statistics by state https://www.nifc.gov/fireInfo/fireInfo_statistics and the Canadian National Fire Database (NFDB, http://cwfis.cfs.nrcan.gc.ca/ha/nfdb).

*Climate*. We used a 1900–2010 global monthly 0.5 × 0.5° gridded terrestrial air temperature and precipitation dataset (v. 3.01, Matsuura & Willmott, Global Climate Resource Pages, Center for Climatic Research, University of Delaware). For the period 1972–2006, 0.5° monthly data were spatially averaged to fit the 1×1° grid and seasonally integrated into winter (JFM), spring (AMJ) and summer (JAS) mean air temperatures and accumulated precipitation.

To estimate the possible responses of area burned under conditions of future climate, we used projected precipitation and air temperature maps for the time period 2010–2039 based on an ensemble of 21 General Circulation Models (GCMs; S1 Table) from the Pacific Climate Impact Consortium (http://pacificclimate.org/) Regional Analysis Tool. We used the SRES AR4 A1B scenario, a moderately optimistic scenario assuming a balance between fossil and non-fossil energy sources conducive to stabilized GHG concentrations after 2050, to explore a mid-range environment for future AAB. We generated a GCM ensemble through bilinear interpolation to a 1x1° grid, and calculated an ensemble mean of GCM projections by first averaging runs within each GCM and then averaging the 21 GCMs (no additional downscaling was performed). Climate projections were given as absolute change compared to average baseline values (1961–1990); projected climate conditions were obtained by adding absolute changes of the ensemble to average baseline conditions.

*Snowpack*. We derived snow cover data from the Northern Hemisphere EASE-Grid Weekly Snow Cover (1966–2007) and Sea Ice Extent (1978–2007) database [50], National Snow and Ice Data Center (NISC), CIRES, University of Colorado, Boulder. Snow cover extent in this dataset is based on digital NOAA-NESDIS Weekly Northern Hemisphere Snow Charts re-gridded to the EASE-Grid (25 km Northern Azimuthal equal-area projection). For the period 1972–2006 we converted weekly snow cover to a single variable, the Last Day of Permanent Snow cover (LDPS). We modified the Natural Resources of Canada definition of median date of continuous snow cover and defined LDPS as the last date included within a period of ≥2 weeks with consecutive snow cover. Negative (positive) LDPS dates applied when the period of permanent snow cover ended before (after) Jan 1^st^ (e.g. February 1 would be LDPS date 32). LDPS were corrected for leap and non-leap years. Between 1972 and 2006, values of LDPS across the entire study area ranged from -58–249 (i.e., Nov 3^rd^ of the prior year to Sep 6 of the fire year). LDPS data were re-projected through bilinear interpolation from the original 25 km EASE Grid to a 1x1° grid, and windowed to 52–170°W, 25–72°N.

### Analytical methods

**Fig 1:**
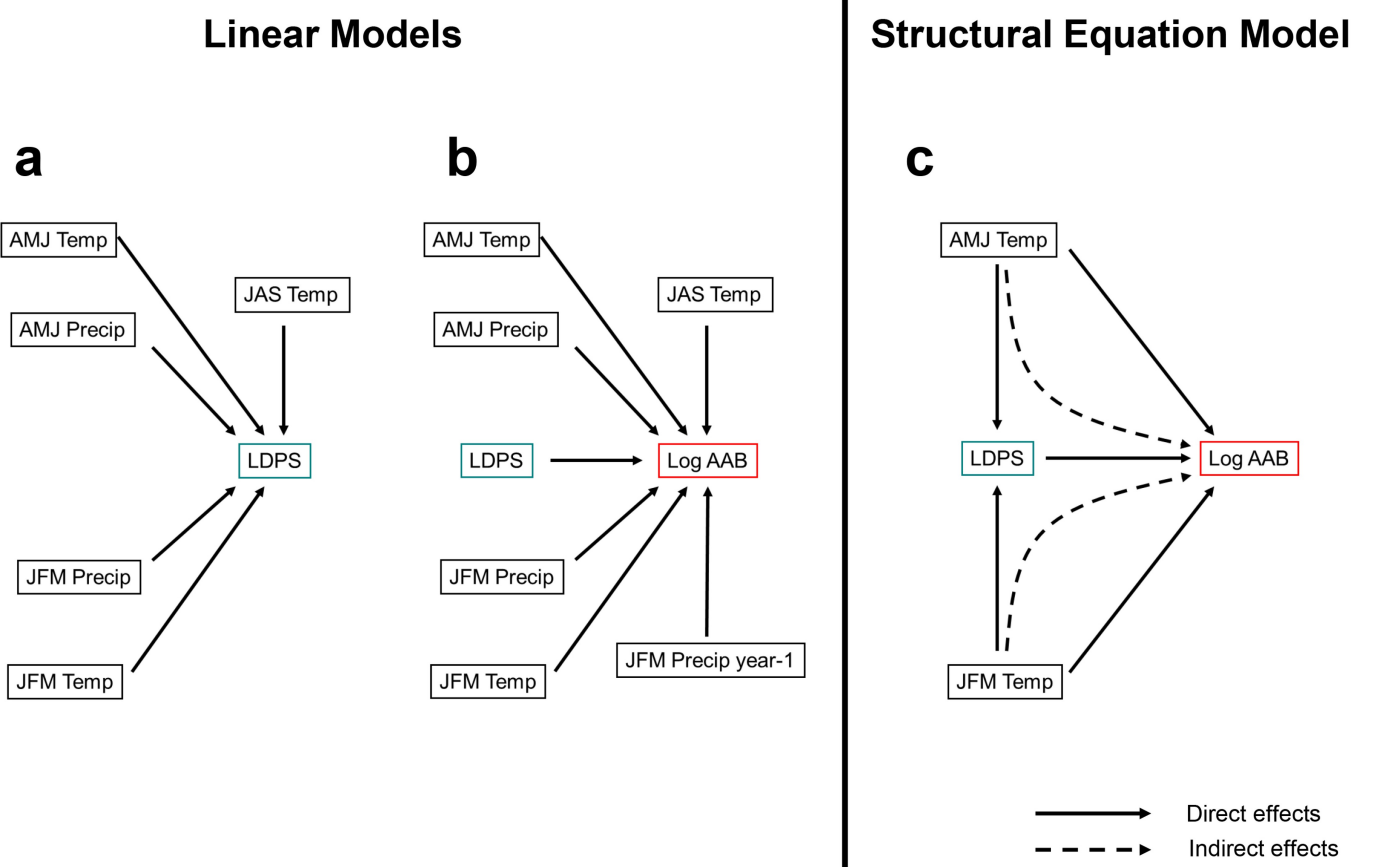
Linear models used for predictions. (a) LDPS (last day of permanent snow cover) and (b) AAB (annual area burned). (c) Structural Equation Model (SEM) diagram of direct and indirect controls on annual area burned in western North America.

To establish the effect of individual forcing variables on log-transformed AAB, we created multiple regression models of log-transformed AAB on all seasonal climate variables and LDPS individually for each grid cell (Fig 1A and 1B), using the linear model function (*lm*) in R [51].

To identify spatial variability in combinations of drivers of AAB over the large geographic domain of this study we performed Principal Component Analysis (PCA) as a singular value decomposition of the correlation matrix of the standardized *z*-scores for each explanatory variable of the regression models. This combined approach created a unique model for each grid cell, which allowed us to isolate combinations of seasonal climate variables and LDPS with the greatest effect on AAB. We used the Theil-Sen robust linear regression method to estimate the magnitude of potential trends in climate, snow cover duration and area burned [52], [53]. The Theil-Sen estimator is the median of the slope of all successive points in the data set, and is relatively insensitive to outliers and more accurate than simple linear regression for skewed and heteroscedastic data [54]. PCA analyses and Theil-Sen trend estimates were performed with the Earth Trend Modeler in IDRISI Taiga (v. 16.05).

Seasonal climate affects fire occurrence both directly (by controlling air temperature and humidity, live and dead fuel moisture, fine fuel production) and indirectly (through control of snowpack duration). Consequently, an analytical method is required that can distinguish direct and indirect effects (that is, whether variables influence a response directly or mediated by another variable) by comparing variance explained via different influence pathways. We used Structural Equation Modeling (SEM; [55]) to evaluate the relative importance of direct winter and spring temperature effects on log-transformed AAB versus indirect effects mediated by snowpack timing. We developed SEM models for winter and spring controls of log-transformed AAB respectively (Fig 1C). Indirect effects were calculated as the product of path coefficients, mapped and compared with direct effect coefficients. Path coefficients were standardized to accommodate the comparison of multiple variables. Standardization involved multiplying the regression coefficient by the standard deviations of corresponding explanatory variables. Unlike some applications of SEM, where a single consensus model is derived, our objective was to quantify the geographically differential influences of seasonal climate and snowpack on AAB. Accordingly, an individual SEM model was built and parameterized for each of the 1562 grid cells in the analysis area. SEM analysis was conducted using the *sem* function of the package = "sem" (v.3.1–5) [56].

LDPS for the A1B scenario was projected using a GCM ensemble of projected temperature (winter, spring and summer) and projected precipitation (winter and spring) on multiple regression coefficients derived from analysis of the baseline period (1972–2004; Fig 1A). Log-transformed AAB was regressed on a cell basis (1° pixel) against same-year seasonal temperatures (winter, spring, and summer), same-year precipitation (winter and spring temperature), LDPS, and previous winter precipitation (Fig 1B). Stepwise backward model selection was performed using the Akaike Information Criterion. We used the function *step AIC* (library *MASS*; [51]) to run a backward elimination on the linear regression until the model with lowest AIC was found. Final models were used to project future log-transformed AAB using seasonal climate variables projected from the GCM ensemble and estimated LDPS (Fig 1B). Cell-based percent changes in AAB were calculated by back-transforming projected log-transformed AAB and comparing with the 1972–2004 baseline median AAB by grid cell and province.

### Model verification

To evaluate model predictions, we compared statistically-projected state/province-level future AAB with our model projections. For each state/province we compared our model-predicted median AAB (2010–2039, scenario SRES A1B) with AAB linearly extrapolated from Theil-Sen trend for 1972–2015. This validation thus included 11 years (2004–2015) not included in the original model calibration and allowed comparison of model projections with empirical AAB trends by region.

## Results

### Trends in seasonal climate and snowcover

**Fig 2:**
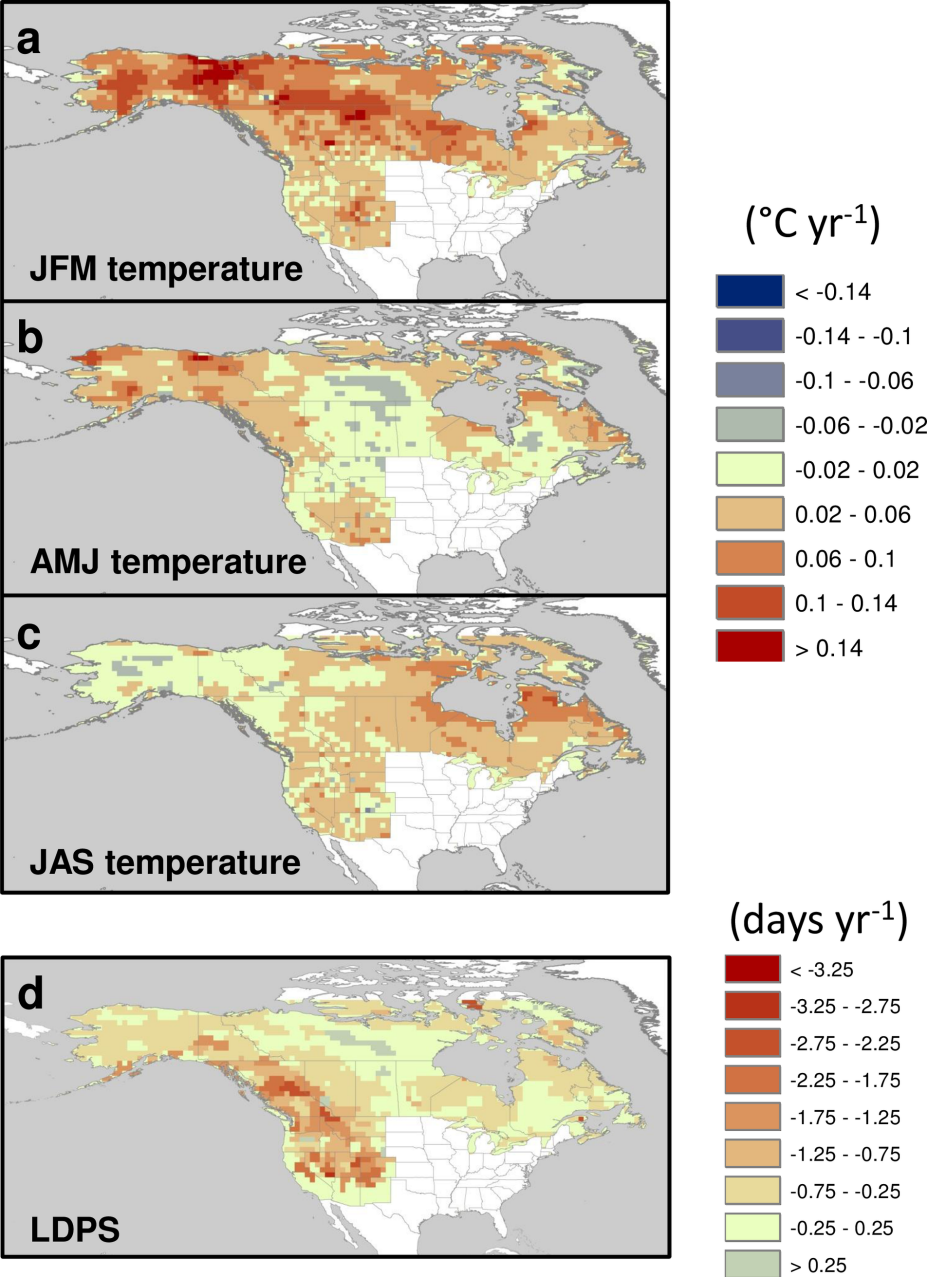
Temporal trends (1972–2006) in instrumental seasonal climate and snow cover duration. (a) Winter (JFM) temperature (°C), (b) spring (AMJ) temperature (°C), (c) summer (JAS) temperature (°C), and (d) LDPS (days/decade), based on the Theil-Sen median slope estimator.

Seasonal climate in western North America has become steadily warmer since the 1970s, and snow duration has decreased steadily (Fig 2).

The most significant trend is in winter (JFM) temperature, with increases > 0.6°C/decade over most high latitudes (Fig 2A). Spring (AMJ) temperatures have increased at rates of > 0.2°C/decade over much of the continent except central Canada and inland Northwest US (Fig 2B). Summer (JAS) temperatures have increased at similar rates but with a different geographic distribution, across most of the western US and central and eastern Canada (Fig 2C). Snow cover duration has decreased since 1972 at an average rate of *ca*. 17 days/decade for most of the interior western US and southwestern Canada (Fig 2D). Shortening of the period of snow cover across the boreal region has been less pronounced, 2 to 7 days/decade.

Trends in regional precipitation were generally less widespread than those recorded for temperature, and of relatively low magnitude (<3 mm yr^-1^; S1 Fig). Statistically (*p* < 0.05) and ecologically (< 15 mm decade^-1^) significant decreases in winter (JFM) precipitation were recorded in Labrador and eastern Québec, northern Alberta, southwestern Alaska, and neutral to small increases in boreal Canada surrounding Hudson Bay. Spring (AMJ) precipitation decreased significantly in southern Quebec, northern Alberta, and parts of north coastal British Columbia and southeastern Alaska, and increased in the northern interior West. Summer (JAS) precipitation decreased significantly across much of the US Northwest, western Alaska, northern Alberta, northern Ontario, and sections of Nunavut and Northwest Territories.

### Spatial variation of drivers of AAB

**Fig 3:**
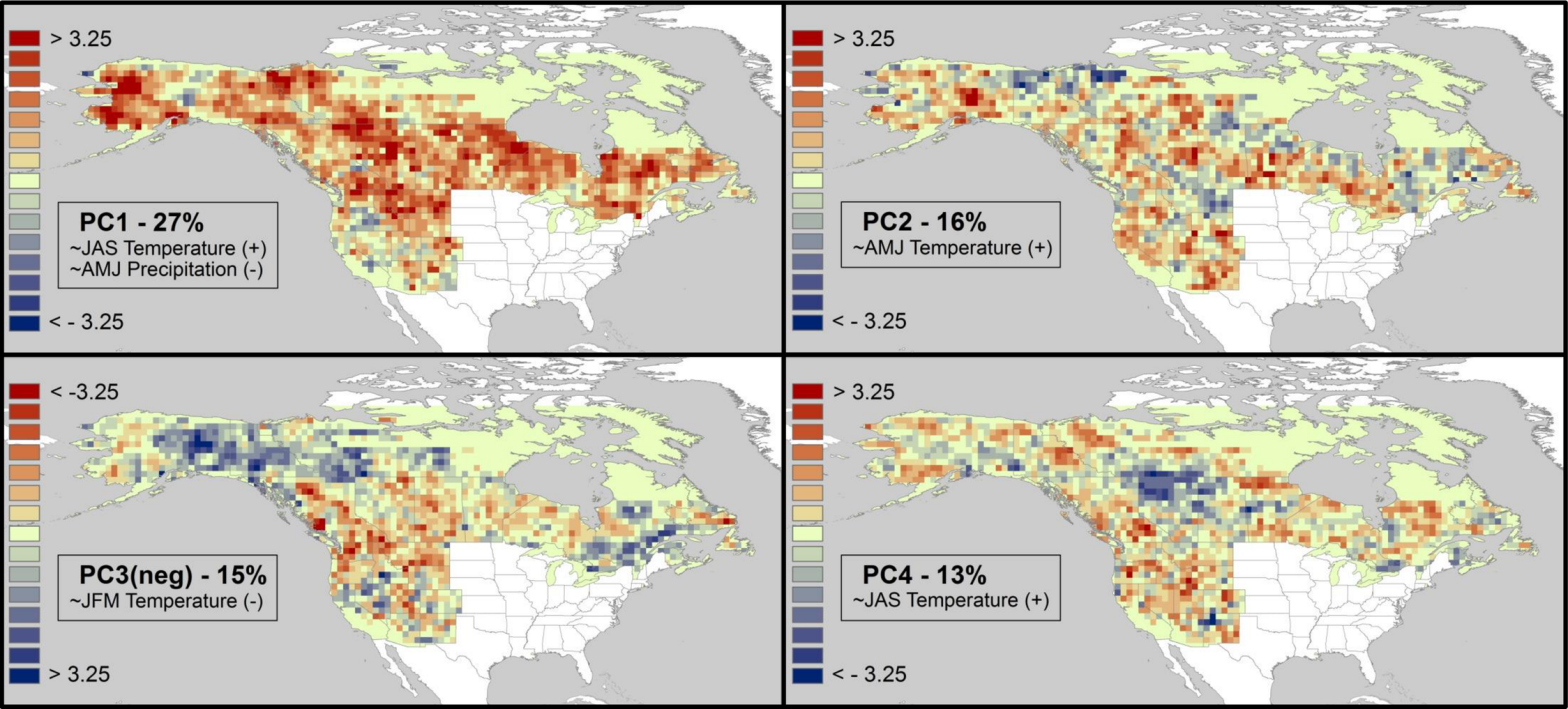
Results of PCA analysis of *z* coefficients of a complete multiple regression model in each grid cell. Panels show the first four principal components and percent in variation in AAB explained. Seasonal climate variables correlated with PC loadings at *r* ≥ 0.5 are listed including the sign of the correlation with AAB. (a) PC1, summer temperature (+) and spring precipitation (-); (b) PC2, spring temperature (+); (c) PC3, winter temperature (-); (d) PC4, preceding year summer temperature (+). Red (blue) colors indicate increases (decreases) in log-transformed AAB with increases in variables correlating positively/negatively with PC scores. Note that PC3 is inverted in sign for ease in interpretation. See S1 Fig for PC5.

PCA analysis of *z*-scores derived from complete cell-based regression models revealed distinct spatial patterns of climate influencing AAB, mostly related to temperature during or before the fire season (Fig 3).

The first principal component (27% of variance in AAB *z*-scores) was correlated positively with summer (JAS) temperature and negatively with spring (AMJ) precipitation. These seasonal variables function as major drivers of spatiotemporal pattern in AAB over most of boreal and western North America, with particular strength over mid to high latitudes (Fig 3A). A second pattern positively related only to summer temperature (PC4, Fig 3D) with influence located at mid-latitudes explained an additional 13% of variance in *z*-scores; both components indicate that increases in summer temperature increased AAB across the continent. *Z*-scores of PC2 were correlated most strongly with increases in spring temperature broadly across the study area (Fig 3B; 16% of variance). PC3 reveals a reversed temperature influence on AAB across high latitudes and eastern Canada, in which colder (milder) winters are correlated with increased (decreased) AAB during the following fire season (Fig 3C; 15% of variance). In contrast, over mid-latitudes, particularly in the northern Rockies, Pacific Northwest, and British Columbia, mild winters are related positively to AAB. Finally, a more regional pattern centered in the Great Basin, Sierra Nevada, with another mode in central and western Alaska (S2 Fig, PC5, 11% of variance) suggests that AAB in these regions is related positively to rainfall during spring of the previous year. In sum, about 82% of variance in AAB *z*-scores is explained by seasonal temperature and precipitation, of which 87% (71% of total variance) is associated with fire-year conditions and the remaining 11% with prior year spring. Summer temperature had the strongest predictive power, followed by spring precipitation, and winter and spring temperature (both positive and negative influences on AAB). Neither snow cover duration nor any other precipitation variables were correlated significantly (*p* > 0.05) to major patterns in *z*-scores of AAB.

### Direct and indirect effects of seasonal temperature and snow cover duration on AAB

**Fig 4:**
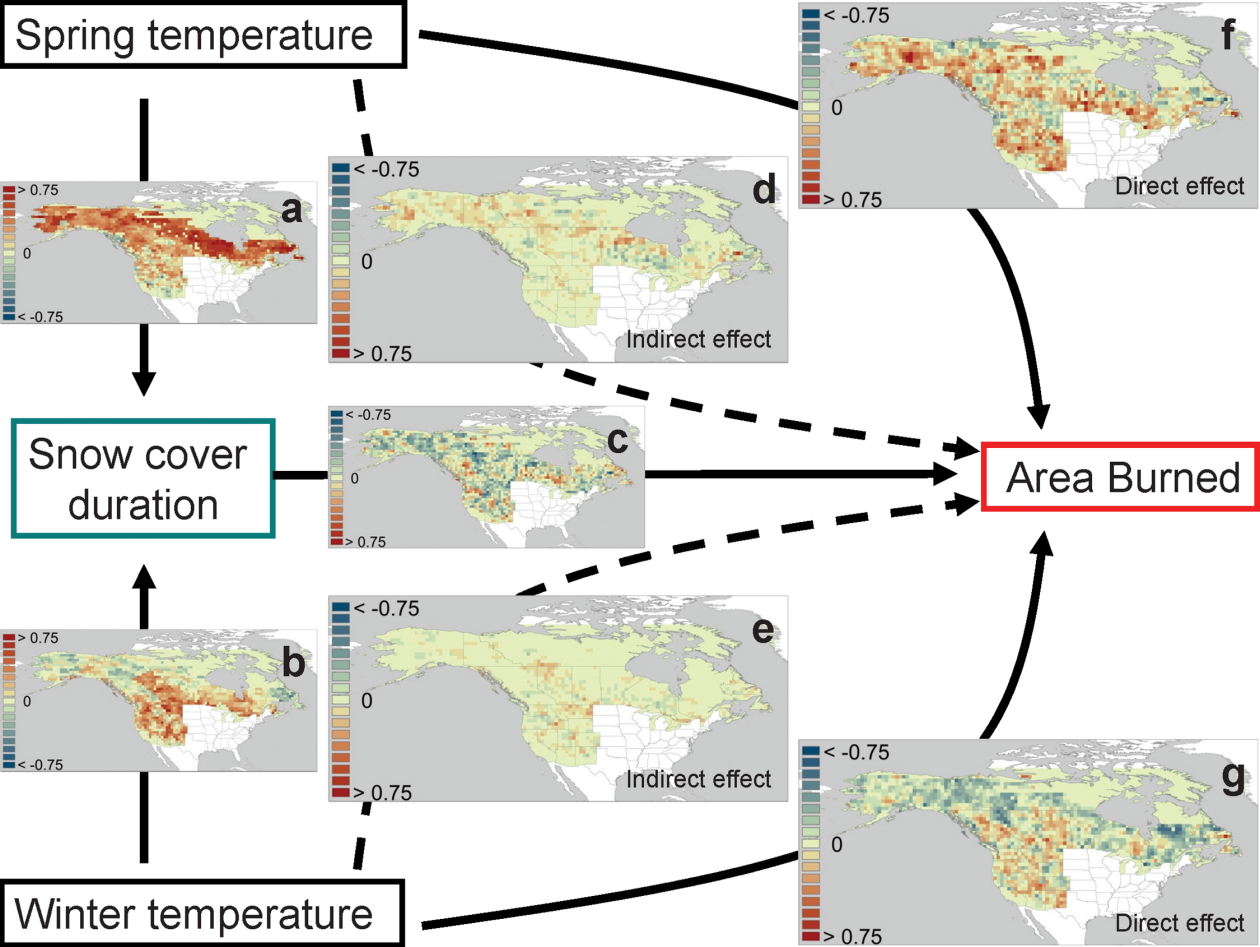
Direct and indirect influences of seasonal temperature and snow cover duration on annual area burned. Results of Structural Equation Models (SEM;1974–2004) show the direct path coefficients for (a) spring and (b) winter temperature on LDPS, (c) the direct effect of LDPS on AAB, the indirect effects of (d) spring and (e) winter temperature on AAB as expressed by variation in LDPS, and the direct effects of (f) spring and (g) winter temperature on AAB.

As expected, warmer temperatures shorten snowpack duration. Both winter (JFM) and spring (AMJ) temperatures had a strong negative (path coefficients < -0.5) influence on snow cover duration, with the former dominating mid-latitudes (western US and across much of southern Canada) and latter higher latitudes across the entire continental boreal zone (Fig 4A and 4B).

**Fig 5:**
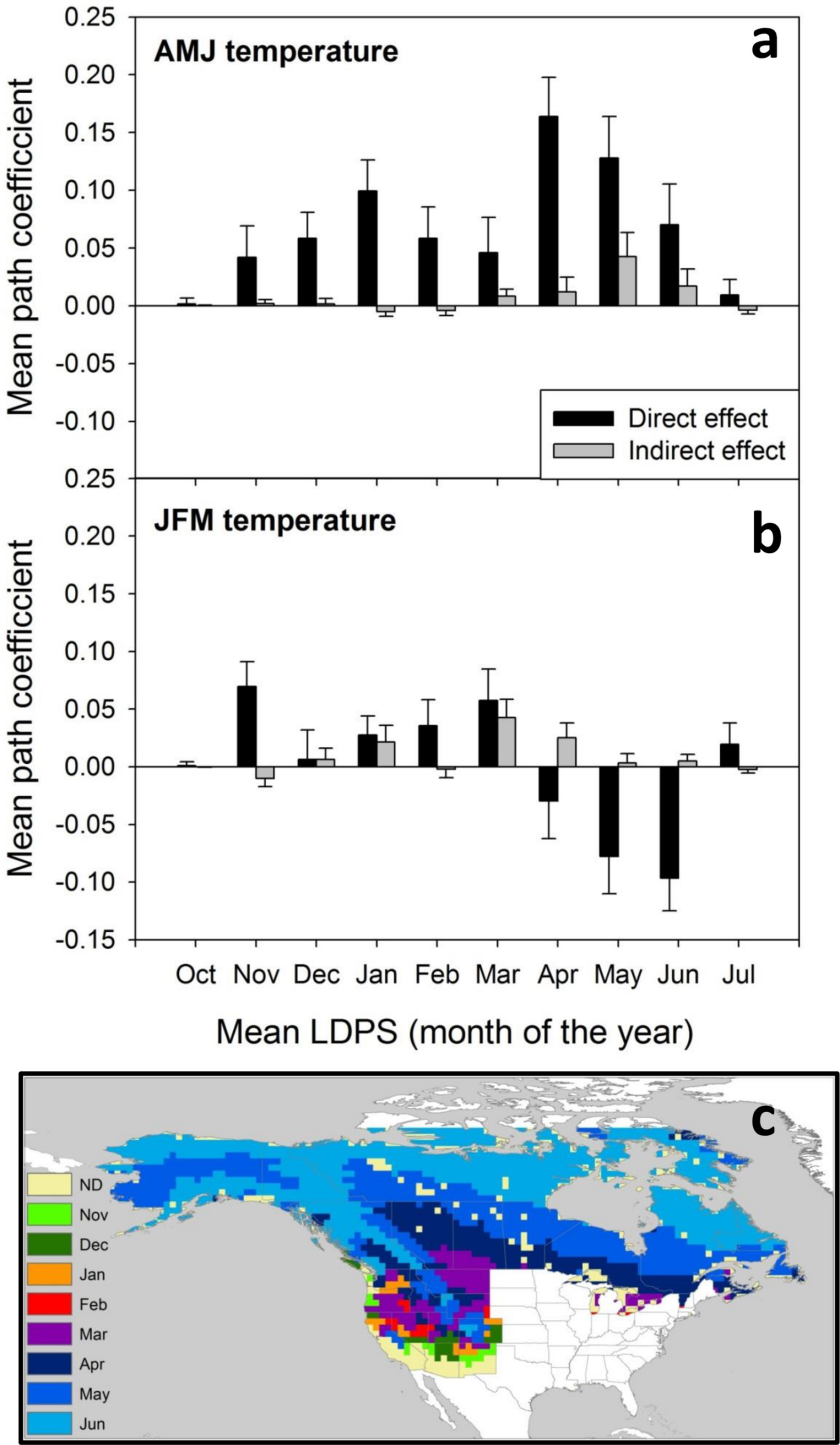
Direct and indirect effects of climate and snow cover on AAB by LDPS regions. Spring (a) and winter (b) mean (± SE) path coefficients averaged over areas of boreal and western North America with areas of similar snow cover duration (monthly classes of long-term (1972–2006) mean LDPS. Black bars indicate direct effects of temperature on log-AAB; grey bars indirect effects on log-AAB mediated by variation in LDPS. (c) Geographic distribution of monthly mean LDPS.

Snow cover duration had a moderate and widespread direct negative influence on AAB with increased influence at higher latitudes and altitudes across the Rocky Mountains (Fig 4C). Despite these transitive relationships (warmer temperatures reduce snow cover duration, which in turn increases AAB), the direct relationship of spring and winter temperatures (Fig 4F and 4G, respectively) was stronger and more widespread than the indirect effect mediated by changes in snow cover duration in most areas (Fig 4D and 4E, respectively). Spring warming-related reductions in snow cover increased AAB only moderately across the northern Canadian provinces and Alaska (Fig 4D). This effect is concentrated in areas with late mean LDPS (Mar-Jun; Fig 5A and 5C).

In contrast, the direct effects of spring warming increased AAB strongly across much of western and boreal North America including the whole western US (Fig 4F), irrespective of the timing of LDPS (Fig 5A and 5C). Effects of winter temperatures were more balanced between direct and indirect effects. Reductions in snow cover duration related to winter temperature caused weak to moderate increases in AAB in higher elevation areas of western US and the Great Plains (Fig 4E), concentrated in areas where LDPS ranges Dec-Apr (Fig 5B and 5C). Direct positive effects of winter warming on AAB were similar in magnitude as indirect effects and were distributed over similar regions of mean LDPS (Fig 5B). Across the northern boreal region (mean LDPS Apr-Jun) winter temperature had a direct negative influence on AAB (Figs 4G and 5B).

### Projections of future climate and AAB

**Fig 6:**
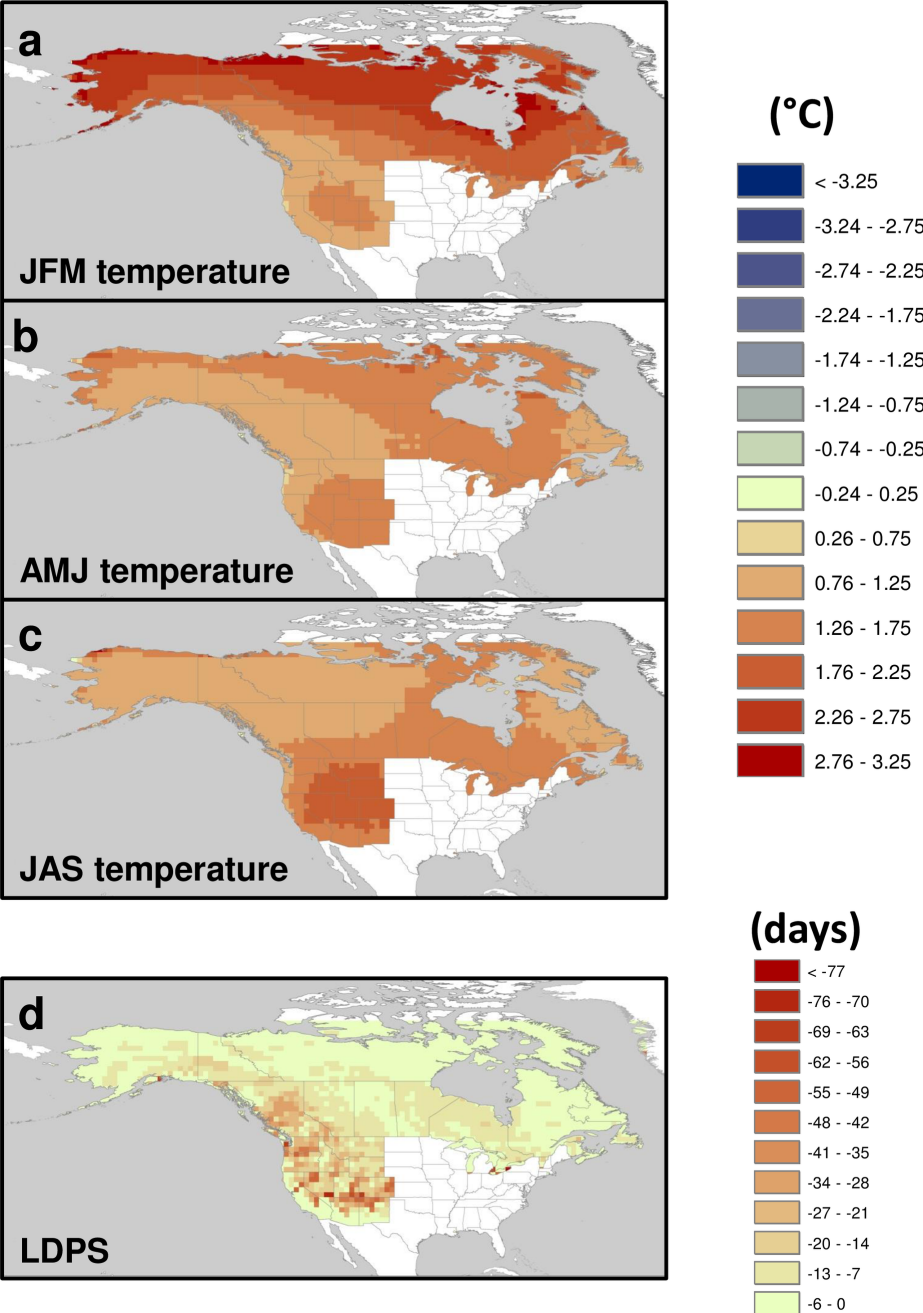
Projected change in seasonal climate variables influencing AAB. Changes are based are for the period 2010–2039 compared to the baseline period 1961–1990 based on an ensemble of A1B emission scenarios: (a) winter (b) spring, and (c) summer temperature (Δ°C); (d) LDPS (last day of permanent snow cover (in Julian date).

Projections of seasonal climate under an A1B scenario for the period 2010–2039 compared to the 1961–1990 baseline (30 year interval) suggest spatially heterogeneous spatial patterns and trends of increases in temperatures and decreases in snow cover duration (Fig 6).

Winter temperature is projected to increase by > 2–3°C over the modeling period (0.4–0.6°C/decade) across northern Canada and Alaska and *ca*. 1.0–1.5°C across the central US (0.2–0.3°C/decade; Fig 6A). Spring temperatures are projected to increase by *ca*. 1.5°C by 2039 (0.3°C/decade) in boreal Canada, northern Alaska and southwestern and central US (Fig 6B), and ~1°C elsewhere. Summer temperatures are projected to increase *ca*. 1.5°C (0.3°C/decade) in eastern Canada, with a higher increase of 2°C (0.4°C/decade) over a core area located in west central US and ~1°C elsewhere (Fig 6C). Snow cover duration (LDPS) is projected to shorten by >50 days (*ca*. 10 days/decade) in some areas of the Colorado Plateau and Intermountain West, and by 25–35 days (5–7 days/decade) across the western US and British Columbia (Fig 6D). Modest decreases in snow cover duration are expected for low elevation and/or high latitude areas of Alaska, northern and eastern Canada (7–20 days = 1.4–4 days/decade).

**Fig 7:**
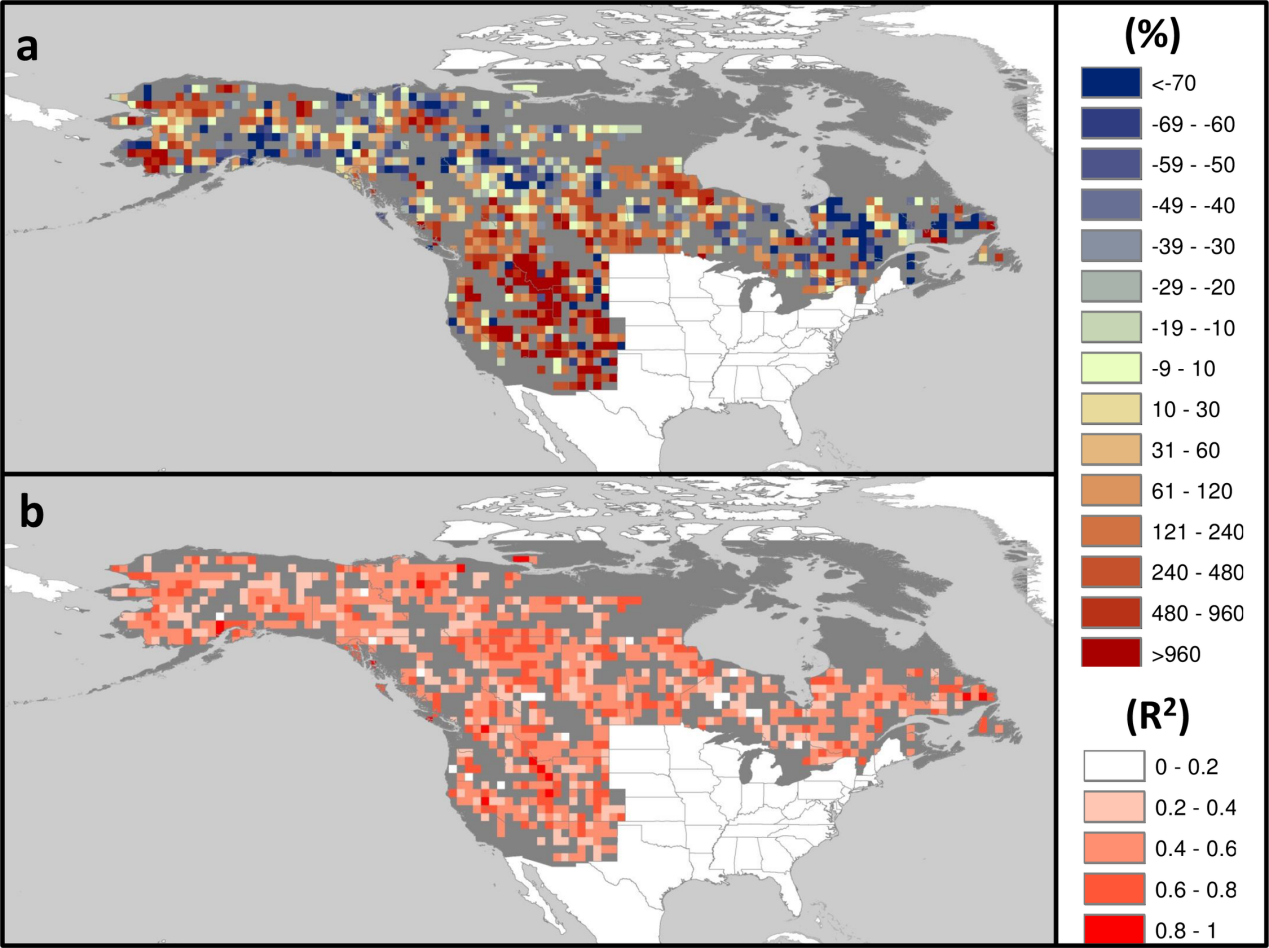
Projected change in AAB. Projections rates are for the period 2010–2039 compared to the period 1961–2004 based on ensemble of A1B emission scenarios. (a) Percent change in AAB resulting from stepwise selection of individual cell-based models, based on AIC model selection criteria; (b) proportion of variance explained (*R*^2^). Only significant models (*p* < 0.05) are plotted.

Of the 1562 cells analyzed, stepwise model selection based on AIC identified 1002 models (64% of all cells analyzed) significant at *p* < 0.05 (Fig 7).

Fifty percent of these models had *R*^2^ > 0.5; *ca*. 20% had *R*^2^ > 0.6 (Fig 7B). Applying these selected models to conditions predicted by the A1B SRES scenario multi-model ensemble (Fig 6A–6D) generates projected percent changes in AAB for 2010–2039 compared to the 1961–2004 baseline period ranging from strongly increasing (one order of magnitude increases in AAB) to moderately decreasing (more than halving of baseline AAB) (Fig 7A). A core of strongly increasing cells (>5x increase in AAB) is located over the northwestern Intermountain US (northern Idaho, western Montana and western Wyoming), central Rockies (central Utah and northern Colorado), southern Rockies and Southwest (New Mexico and northern Arizona), and western Nevada. Other areas with important (>2x) increases in projected area burned include the Sierra Nevada in California, the Cascade Range in Oregon and North Cascades in Washington, western Alaska and northeastern Manitoba. Less pronounced increases (50–100%) in AAB are projected for southern British Columbia, central Alberta, south-central Saskatchewan and southern Manitoba. Decreases (>50%) in AAB are predicted for some areas of central Québec, northern Alberta, western Southwest Territories, and parts of Yukon and south-central Alaska.

**Fig 8:**
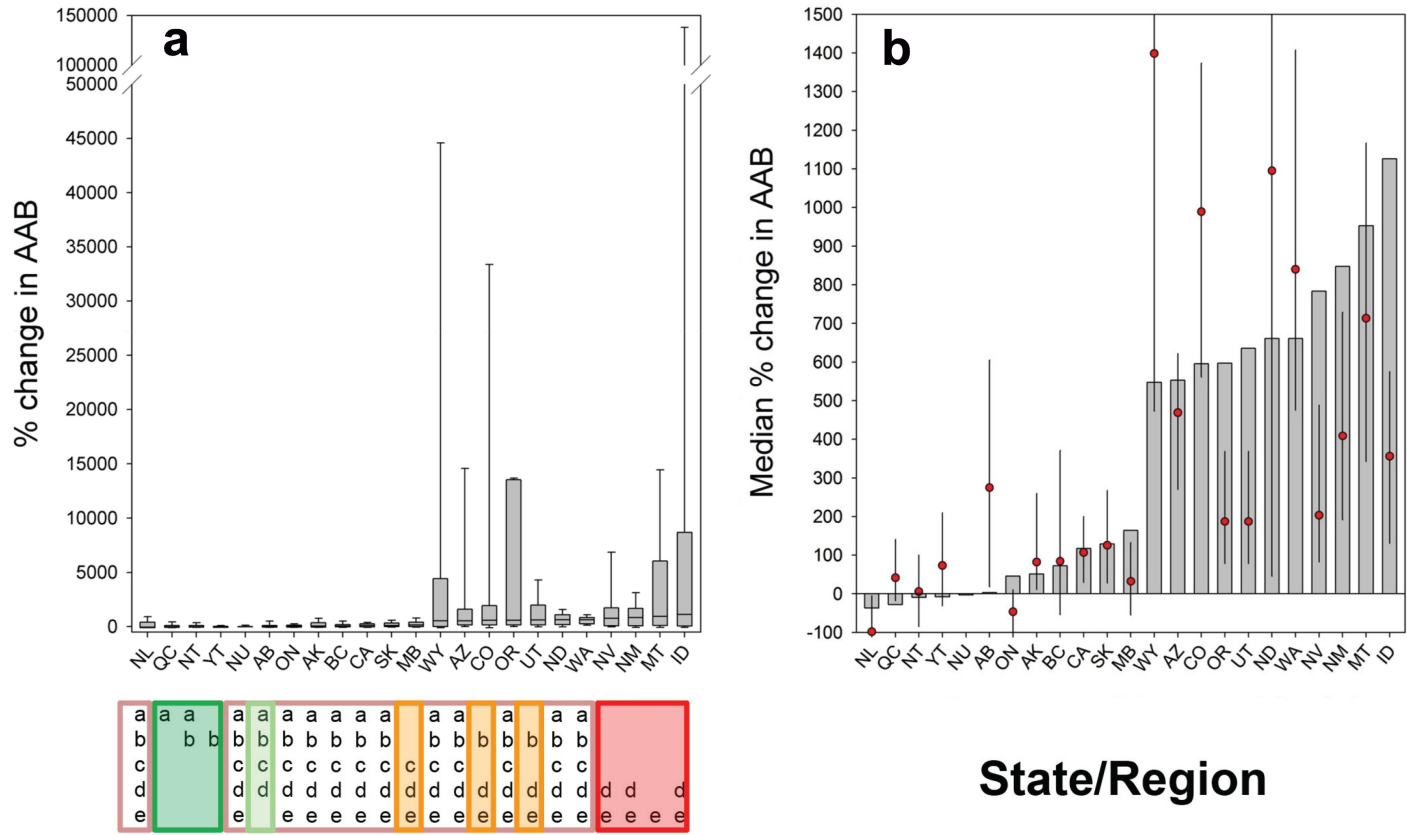
Changes in AAB across United States and Canada by state/province. (a) Boxplot of percent change in AAB (2010–2039 *vs*.1961-2004, SRES A1B scenario) binned by US state or Canadian province, based on significant models with *p* < 0.05. Sharing of any letter (below the graph) indicates lack of significant differences in medians of percent change in AAB based on Bonferroni-corrected *a posteriori* comparisons of a Kruskal-Wallis median test. Colored boxes indicate groups of states/regions with statistically similar medians ordered from low (green) through high median values (red). (b) States/regions ordered by increasing median change in AAB. Histograms are model projections based on the 1976–2006 baseline period; red dots are extrapolated increases in median AAB and bars are 95% confidence intervals estimated from Theil-Sen trends (1972–2015).

Median percent changes in AAB predicted for the period 2010–2039 calculated over states and provinces show three areas of distinct increased fire activity projections (Fig 8A and 8B).

Eleven US states (ID, MT, NM, NV, WA, ND, UT, OR, CO, AZ, WY,) show >5x median increases in AAB. Median increases in excess of 700% are predicted for ID, MT, NM and NV, and strong upper quartile increases are predicted for OR, ID, MT, and WY. Moderate (*ca*. 10–150% median) increases in AAB are projected in seven states or provinces (MB, SK, CA, BC, AK, ON); net decreases in AAB are projected in five provinces (NU, YT, NT, QC, NL).

Comparison of these projections against empirical data indicates that the model successfully captured the primary trends in AAB and its respective drivers (Fig 8B, red dots and error bars). Not surprisingly, empirically-based projections were noisier than model output, reflecting the contingent nature of large wildfires. Nonetheless, AAB in all states and provinces projected to experience ≥ 100% increase in wildfire under the baseline model is increasing as projected; AAB is increasing more slowly in some states (OR, UT, NV, ID) than model projections, and more rapidly in others (AB, WY, CO, ND, WA). With the exception of AB, states and provinces with projections of more moderate AAB increase (NL, QC, NT, YT, ON, AK, BC, CA, SK, MB) matched model projections closely. Boreal regions all show negative or neutral (1972–2015) trends, consistent with our projections.

## Discussion

Seasonal climate variation exerts a primary control on the length, location, and intensity of fire seasons in western North America and worldwide. The amount of area burned by wildfires represents a complex integration of productivity, ignition patterns, landscape configuration (i.e., fuel connectivity), synoptic and local weather, and seasonal climatic conditions that condition fuels and influence fire spread and the length of fire season, along with the important role of anthropogenic ignitions [18], [20], [32]. Seasonal climate also regulates interannual variation in fuel production and fuel moisture, which act as primary proximate regulators of fire extent. Interactions of these factors produce highly heterogeneous responses at sub-continental to continental scales.

Our seasonalized climate and snowpack model is clearly an oversimplification of the climate-vegetation-fire system, but it allows us to evaluate the relative influences of various drivers of fire activity over a heterogeneous region, given the data available [57]. Because our model does not incorporate vegetation feedbacks we restricted our projection to the immediate future decades, allowing that more substantial shifts in vegetation may occur over longer (centennial) time [35], [58]. In accordance, these wildland fire area projections should be interpreted as near-term (multi-decadal) responses to climate system variation, recognizing that climate trends themselves (seasonal temperature and precipitation) do not progress linearly over time.

Our fire activity analyses and projection modeling indicate two contrasting responses across the sub-continent: (1) areas of strong increasing trends in burned area (>1.5% yr^-1^) and strong predicted increases in future AAB (>500% median AAB compared to the baseline period) and (2) areas of more moderate change: no significant trends in AAB during the last four decades and prediction of modest increases (<100% in median AAB) to modest decreases (<50% in median AAB). Despite strong directional climatic trends and high predicted rates of warming across northern and boreal North America, annual burned areas by wildfires during the upcoming decades is likely to respond in a geographically heterogeneous fashion, reflecting interacting environmental and seasonal drivers that control wildfire occurrence and spread [46], [59].

These geographically contrasting responses of AAB to warming climate reflect different combinations of factors related to antecedent (cold season) and proximate (fire season) climate that control fire occurrence and spread, and thus ultimately modulate AAB. Consistent with literature linking temperature to fire activity [22], [26], [27], [60] our analyses suggest that warm season (summer and spring) temperature is the dominant influence of AAB over large portions of the sub-continent across nearly all of forested Canada and the interior northwestern US (Figs 3 and 4). The most widespread control of AAB is summer temperature, which (partly in conjunction with a spring drought effect) significantly and positively influences AAB, accounting for *ca*. 40% of the variability in *z* scores of most cell regression models. Summer and spring temperature jointly drive the largest proportion of spatial variability in change in AAB across nearly all of forested Canada and the interior northwestern US (Fig 3). Elevated winter temperatures are associated with decreased AAB in northern Canada, eastern Alaska, and Quebec, and increased AAB in the US Pacific Northwest, British Columbia, and across southern and central Canada. This seasonal variable may exert the strongest influence on AAB in climates with shorter growing and fire seasons, where high summer evaporative demand is the main driver of fuel desiccation. Other plausible indirect mechanisms related to summer warming in these higher latitudes reflect increased convectivity and resulting elevated probability of lightning fires [61], and promotion of insect attacks, which can generate quantities of dead fuels as well as large areas of drought-stressed fire-prone forests across landscapes, although effects of insect outbreaks on fire severity and area burned are highly variable [62], [63]. Summer temperatures exert mixed control on AAB in warmer mid-low latitude ecosystems (e.g. semiarid mountains of southwestern US) where fuel desiccation below critical thresholds occurs in late winter or spring, and where monsoonal activity can mitigate the influence of summer temperature fire activity.

Spring and winter temperatures have partially overlapping positive effects on AAB with summer temperature across the continent, but with larger influence over mid-latitudes. Spring and winter temperatures can be important controls of critical fuel moisture levels in ecosystems with longer growing seasons where plants start to actively metabolize and exchange gases with the atmosphere well before summer. In these systems, increased late winter and/or spring temperatures can induce moisture stress in plants, dropping fuel moisture levels below critical flammability points by the time fire season starts. This lagged effect may occur and accumulate up to several months before the fire season even starts, particularly in warmer climate regimes (e.g. southwestern US; [29]).

Snowpack duration has been proposed as a mechanism controlling wildfire AAB related to late winter/spring warming [27, 38]. Earlier snowpack melt can increase wildfire activity because the continuous replenishment of water into the soil by snowmelt is eliminated during the pre-fire season, thus allowing fuel to reach critical desiccation levels that promote fire. Change in area burned in the interior western US is associated with areas of significant decrease in LDPS, but many areas (eastern Montana and Wyoming, southern Saskatchewan and Alberta, Alaska) are predicted to increase in burned area despite small or non-significant change in LDPS (Fig 4). LDPS was not correlated significantly (*p* > 0. 05) with any of the first five principal components of variation in area burned, independent of summer temperature, spring temperature and precipitation, and winter temperature. Snowpack duration responds to these same variables and is thus correlated with the underlying mechanisms that govern variation in area burned.

Our SEM analyses indicate that winter and spring warming produce major sub-continental-scale alteration in snow cover duration. Snow cover duration can also have an important (negative) overall influence on AAB (Fig 4). Yet, when partitioning direct versus indirect effects of spring and winter temperatures, the effects of temperatures mediated by snowpack duration have a weaker overall effect than the direct effect of warmer temperatures on AAB. While direct effects of spring temperature on AAB spread across the entire continent, along areas with snowpack disappearance timing ranging from November (Southwestern US) through June (northern boreal and higher elevation areas), the indirect effect of spring snowmelt is less than a third in strength and is restricted to areas where on average snowpack disappeared around May (encompassing most mid-high elevation forests in the Rockies and areas of the southern boreal shield areas and interior Alaska; Figs 4C and 8). Similarly, indirect effects of winter temperatures altering snow cover duration have an important influence only in areas where snow cover disappears on average in March (lower elevation mid-latitude intermontane areas, plateaus and plains). This suggests that inter-annual snowpack duration variability may control regional AAB primarily within certain altitudinal or latitudinal belts.

Our results are consistent with those of Westering and colleagues [27] based on seasonal snowpack data, which suggest that the greatest effects of earlier spring snowmelt in the US occur at an altitudinal belt centered at around c. 2100m in the northern Rockies. AAB in colder forests (high elevations or latitudes) with normally continuous snow cover during the spring may not be altered as strongly by variations in earlier summer snowmelt during the modeling period in this study (to AD 2039). Conversely AAB in forests with warmer climates (lower elevations or latitudes) normally not covered by snow in spring may be less affected by variations in the timing of late winter snowmelt compared to the direct effects of temperature. Winter temperature, a seasonal variable used less frequently for predicting fire risk or activity, showed geographically distinct relationships with AAB. At lower latitudes (upper western US, south-central and southwestern Canada), winter temperatures relate positively to area burned, suggesting the effects of both direct effects on fuel desiccation or modification of snowmelt timing.

Our forward projections are decadal in temporal extent (to 2039), in contrast to some other modeling efforts that project to the end of the 21^st^ century. Given that the largest relative rate of cold season warming during our time frame is predicted to occur at higher latitudes (Fig 6A), it is in this region where the changes in AAB predicted solely by fire season variables may be tempered (producing neutral responses) and in some cases counterbalanced by the effects of cold season temperature over the coming decades.

We found highly variable outcomes across the boreal forest zone, indicating complex controls on area burned (Fig 7A). Boreal fire regimes are affected strongly by seasonal temperatures because sufficient fuel mass is generally present; thus, these fire regimes are characteristically energy- and not moisture-limited with respect to fuel mass and flammability, although interannual variation in biomass production may be more significant in some areas than previously understood [32]. Over time, the majority of the area burned in the boreal forest is the result of large, infrequent fires that occur during extended periods of high pressure systems that result in rapid fuel drying [23], [60], [64]. Consequently, most area burned in North American boreal forest is the result of a relatively small number of very large fire years, which are determined strongly by short-term (days to weeks) meteorological events driven by episodic anomalies in atmospheric circulation, and less so by annual patterns of seasonal-scale temperature *per se* [64].

The blocking highs and ridges in atmospheric circulation (typically 500 mb geopotential heights) that create hot dry weather for 1–2 weeks are associated with phases of PDO/ENSO, AO, and the Pacific North American (PNA) pattern of atmospheric circulation. Duffy and colleagues [65] found that 69% of AAB in Alaska occurred when winter PDO was negative, which indicates correlations with spring precipitation. Hartmann & Wendler [66] found strong negative correlations of winter PDO with winter temperature. Negative phases of PDO correspond to a weakened Aleutian low and low pressure over continental North America; the resulting meridional flow of arctic air, with low cloudiness, may increase winter drought effects and generate larger fires. Our seasonal-scale model may understate the role of these shorter-term dynamics.

Projected changes in AAB for western and boreal North America are in agreement with recent global assessments of future fire activity derived from satellite data, which predict a geographically variable and heterogeneous redistribution of fire across continents and the global [3], [7], [8], [12], [20], [32], [67]. Our models suggest that, at least in the short term (decades, our model time frame), warming will produce strong increases in wildfire AAB in mid latitude temperate forests of western North America but more variably across boreal forests; these projections were validated with area burned data through 2015 (Figs 5 and 8). Similar geographically variable responses of fire activity have been shown by other studies using different methodologies. Projections of fire weather for a 2xCO_2_ scenario using monthly [68], and daily [12], [20] weather variables show differences in predicted responses between central North America (increased fire weather) and eastern/northwestern Canada (decreased fire weather). In a global analysis using multivariate statistical generalized additive models combining existing fire occurrence, climate, net primary productivity, and ignition data for North America, Krawchuk and colleagues [7] found increases in fire activity over the central mid-latitudes of the continent and decreases in fire activity towards boreal regions both in the northeast and northwest of North America. Moritz and colleagues [3], using statistical modeling of drivers of fire probability, project increased fire occurrence (although not AAB) for western US, south-central Canada, and northern Alaska with likely decreases in fire activity in central boreal Canada and Alaska until mid-century (2010–39, similar to our modeling frame), and then increased fire probability across much of western and boreal North America in the later 21^st^ century. Parisien and colleagues [32] found highly heterogeneous drivers of area burned across Canada using both annual and multi-decadal average models.

Our results are consistent with other projections of climate-altered fire activity in western North America. Littell and colleagues [28] predicted increases in median AAB of 833% for forested areas of the Blue Mountains, OR by 2040 under the A1B scenario, with lower increases (190%) in other mountain ranges such as the Western Cascades. Variability in projections among US states is similar to that reported by McKenzie and colleagues [14] where the strongest positive increases in AAB are expected for MT, AZ, NM UT and WY, and slight projected decreases in AAB in CA, similar to our results. Longer term retrospective studies suggest that, despite a warming trend during the 20th century [69], the frequency and size of fires in the boreal forest region is likely to remain highly variable [47], [70].

Our results for the boreal forest projections contrast with some similar statistical approaches using relationships between climate and historical AAB. Flannigan and colleagues [71] predicted overall average increases in AAB of 76–152% by the end of the 21^st^ century, although they found significant variability among ecozones, with the greatest increases in more northern and westerly ecozones. This discrepancy is most likely attributable to their longer modeling time frame and more extreme climate scenario (3×CO_2_ GCMs), whereas our predictions are limited to the next few decades under a more moderate climate future. Abbott and colleagues [5] projected increased pyrogenic CO_2_ emissions by 2100, but a considerably smaller increase under moderate emissions scenarios at 2040.

Analyses of relationships between seasonal weather, vegetation, and fire activity trends under current and future climate scenarios have been performed using a variety of approaches (mechanistic dynamic vegetation models, statistical models) with different sets of surface climate variables (fire danger ratings, meteorological data), temporal resolutions (daily, monthly, seasonal data), spatial resolutions (global, biomes, ecological zones, countries, regions, states, grid cells or hexels (generally < 1°)) and fire activity response variables (fire weather, annual area burned, fire occurrence, energy release component, fire severity). This modeling variability may contribute to differing projections of how fire responds to suites of interacting parameters of climatic variability and ecological response over broader geographic regions and scales, including the inherent difficulty of modeling outside the range of historical climate for a given ecosystem.

As with most forward modeling studies, our approach projects current relationships of AAB to seasonal climate onto climates outside the reference period. Such extrapolation is an inherent source of potential error in any modeling study of future climate and ecological responses [60], [71], [72]. Our projections conform to the IPCC definition of “near-term” [72], the period during which projections are most strongly influenced by the initial state (here, the baseline period 1972–2006). Non-linear and possibly threshold responses of vegetation, seasonal climate, and fire to global climate regimes are all possible, as are altered feedback interactions between the biosphere and the climate system [7]. Here, we focused on a suite of seasonal climate and snowpack factors, but we acknowledge that other variables in the climate system could play a role. In addition, area burned in smaller fires (200–400 ha) in some land ownerships may be incompletely reported in the early period of record (pre-1980), although there is no indication that this would have a significant effect on regional area burned over the entire period and geographic extent of the current study. All of these factors could influence the accuracy of these projections, especially beyond the modeling period stipulated here [3]. Comparison of these results with other studies must consider differences in study design, including emission scenario and GCM(s) employed, spatial scale (grid cell size and spatial extent), baseline time period and period of future simulation, among others [32]. Climate projections under various emissions scenarios diverge less strongly to 2040, with greater differences emerging later in the century, the characteristic uncertainty inherent in any GCM-based projection [73].

Fire severity–the effects of wildland fire on ecosystem components such as soils and vegetation–is an implicit variable in this study and is not quantified here. Historically, large burned areas cannot necessarily be equated with high fire severity. Indeed, the fire scar record indicates the opposite: large areas of many forest types in western North America experienced spatially widespread, low to moderate severity fires over many centuries [74–77]. Our projections of increased (decreased) AAB into the mid-21^st^ century do not necessarily imply that the proportion of burned area affected by high severity will change, although increases in severity have been detected in some regions in recent decades [36], [78]. Some ecological responses to fire, for example rates of overstory tree mortality, are increased by environmental factors such as drought stress [39]; if these relationships hold, then larger burned areas could also include increased areas of tree mortality, with cascading ecological effects.

### Implications for management and policy

Regional variation in annual area burned represents an important metric for land managers and policy makers. Strong seasonal climatic controls on AAB suggest that increases in future burned area in some regions may be difficult to offset with management actions such as fire suppression activities, which tend to be less effective under extreme fire weather conditions [79]. Fire-induced tree mortality will also interact with climatic stressors, particularly temperature-related soil moisture deficits which are projected to become chronic and extreme by mid-century throughout much of western North America [26], [80], [81]. Combinations of landscape-scale fires with large, contiguous high-severity patches and rapid increases in temperature may set the stage for widespread conversion of current forest to shrubland and other more xeric-adapted community types in some ecoregions [82], [83]. Fire can accelerate or alter such vegetation change by triggering such “tipping point” ecosystem responses; larger AAB multiplies this process over larger areas, potentially accelerating the rate of vegetation conversion under background of changing climate [84]. If this occurs, managers may be even more hard pressed than currently to maintain ecosystems in anything approaching their current structure and condition. Area burned is also potentially an index of regional fluxes of carbon to the atmosphere, and thus an important feedback to the climate system by potentially increased CO_2_ emissions [1], [5], [42], [85], and by accelerating ecosystem conversions to functional plant types that sequester less carbon per unit area [86].

Understanding the effects of changing climate on ecosystem processes and atmospheric fluxes at continental to global scales has become an urgent need. Wildfire is a global Earth system process that both integrates and influences many other interactions between ecosystem and the climate system. Fire mediates other ecosystem responses to changing climate, for example by modulating forest density and composition, and thus providing a mechanism by which ecosystems adapt to changing climate conditions. Uncertainties in key elements of climate projections could be compounded by nonlinear responses of fire to climate variability. Fires may also act as triggers for abrupt and irreversible change to novel configurations under future climate [44], [83]. As climate change progresses, the projected changes in the area affected annually by fire may be an important multiplier of these effects in coming decades.

## Supporting information

S1 Fig Temporal trends in instrumental seasonal precipitation (1972–2006). Left panels: trend magnitude based on Theil-Sen median slope estimator for JFM, AMJ, JAS respectively. Right panels: trend significance based on Mann-Kendall test. Cool colors indicate increasing precipitation; warm colors indicate decreasing precipitation.
(TIF)

S2 Fig Spatial correlation of PC5 (preceding year spring temperature, positive correlation with AAB, 11% variance in AAB explained) from PCA analysis of *z* coefficients of a complete multiple regression model in each grid cell. Red (blue) colors indicate increases (decreases) in log-transformed AAB.
(TIF)

S1 Table General Circulation Models and runs used for the A1B emission scenario ensemble. (DOCX)
