# Supplementary figures and images for: Direct and indirect climate controls predict heterogeneous early-mid 21st century wildfire burned area across western and boreal North America
Source: PLoS One. 2017 Dec 15;12(12):e0188486. doi: 10.1371/journal.pone.0188486 (PMC5731736; doi:10.1371/journal.pone.0188486)

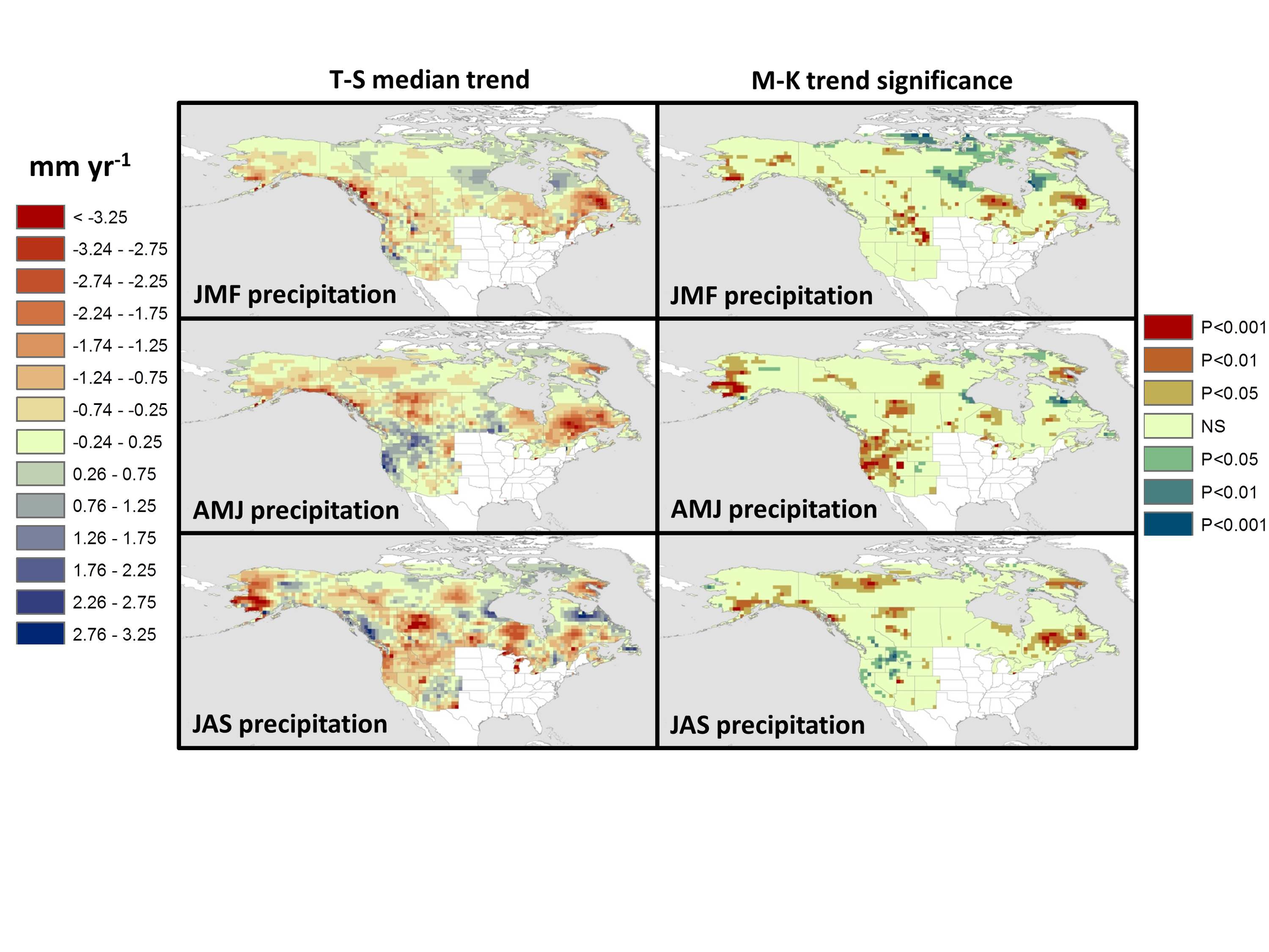

Supplement: S1 Fig — Left panels: trend magnitude based on Theil-Sen median slope estimator for JFM, AMJ, JAS respectively. Right panels: trend significance based on Mann-Kendall test. Cool colors indicate increasing precipitation; warm colors indicate decreasing precipitation. (TIF) [file pone.0188486.s001.tif]

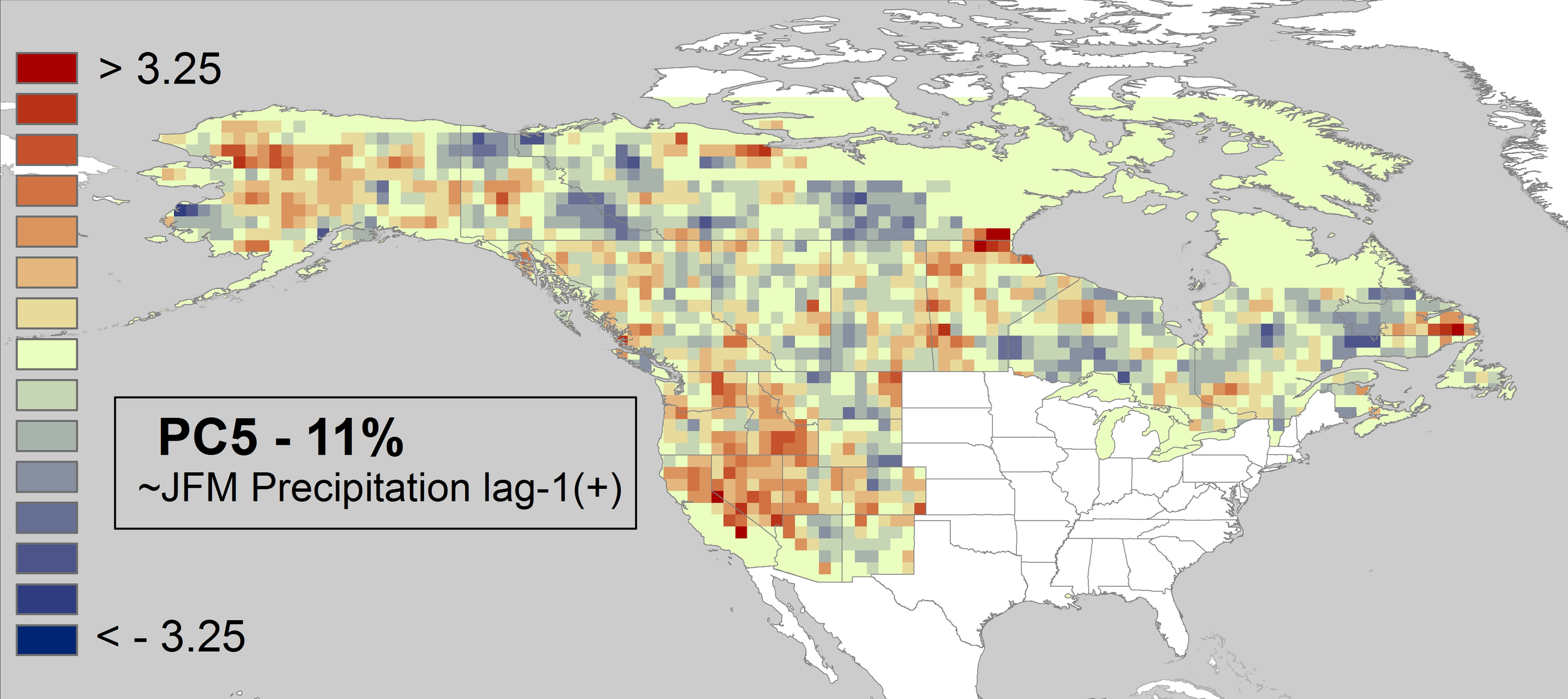

Supplement: S2 Fig — Red (blue) colors indicate increases (decreases) in log-transformed AAB. (TIF) [file pone.0188486.s002.tif]
