# Supplementary material for: Direct and indirect climate controls predict heterogeneous early-mid 21st century wildfire burned area across western and boreal North America
Source: PLoS One. 2017 Dec 15;12(12):e0188486. doi: 10.1371/journal.pone.0188486 (PMC5731736; doi:10.1371/journal.pone.0188486)
Supplement: S1 Table — (DOCX) [file pone.0188486.s003.docx]

| **GCM acronym** | **Runs** | **Source** |
| --- | --- | --- |
| BCCR_BCM2.0 | 1 | Bjerknes Centre for Climate Research |
| CCCMA_CGCM3 | 1 | Canadian Centre for Climate Modelling and Analysis |
| CCCMA_CGCM3_T63 | 1 |  |
| CCCMA_CGGEOREF_CNRM_CM3 | 1 |  |
| CSIRO_MK30 | 1 | Commonwealth Scientific and Industrial Research Organisation, |
| GFDL_CM21 | 1 | Geophysical Fluid Dynamics  Laboratory |
| GISS_AOM | 2 | Goddard Institute for Space Studies, NASA |
| GISS_EH | 3 |  |
| GISS_ER | 5 |  |
| IAP_FGOALS10G | 3 | Institute of Atmospheric Physics, Chinese Academy of Sciences |
| INMCM30 | 1 | Institute of Numerical Mathematics, Russian Academy of Science |
| IPSL_CM4 | 1 | Institut Pierre Simon Laplace |
| MIROC32_HIRES | 1 | Center for Climate System Research, University of Tokyo |
| MIROC32_MEDRES | 3 |  |
| MIUB_ECHO-G | 3 | Max Planck Institute for Meteorology |
| MPI_ECHAM5 | 4 |  |
| MRI_CGCM232A | 5 | Meteorological Research Institute, Japan Meteorological Agency |
| NCAR_CCSM30 | 8 | National Center for Atmospheric Research |
| NCAR_PCM1 | 3 |  |
| UKMO_HADCM3 | 1 | Hadley Centre for Climate Prediction and Research |
| UKMO_HADGEM1 | 1 |  |
